# High-resolution microbiome analysis reveals exclusionary *Klebsiella* species competition in preterm infants at risk for necrotizing enterocolitis

**DOI:** 10.1038/s41598-023-34735-2

**Published:** 2023-05-16

**Authors:** Spencer Coleman, Katrin Unterhauser, Karim Rezaul, Nagender Ledala, Stephanie Lesmes, Melissa J. Caimano, Yanjiao Zhou, Eric Jackson, Dawn Gratalo, Mark D. Driscoll, Adam P. Matson

**Affiliations:** 1 Department of Pediatrics, UConn Health, Farmington, CT USA; 2 Present Address: University of Connecticut School of Medicine, 263 Farmington Avenue, Farmington, CT 06030 USA; 3 Department of Research, Connecticut Children’s Medical Center, Hartford, CT USA; 4 Department of Medicine, UConn Health, Farmington, CT USA; 5 Department of Molecular Biology and Biophysics, UConn Health, Farmington, CT USA; 6 The Jackson Laboratory for Genomic Medicine, Farmington, CT USA; 7 Intus Biosciences, Farmington, CT USA; 8 Division of Neonatology, Connecticut Children’s Medical Center, Hartford, CT USA; 9 Department of Immunology, UConn Health, Farmington, CT USA

**Keywords:** Bacteria, Clinical microbiology, Microbial communities, Paediatric research, Translational research, Gastrointestinal diseases, Infection

## Abstract

Intestinal colonization with *Klebsiella* has been linked to necrotizing enterocolitis (NEC), but methods of analysis usually failed to discriminate *Klebsiella* species or strains. A novel ~ 2500-base amplicon (StrainID) that spans the 16S and 23S rRNA genes was used to generate amplicon sequence variant (ASV) fingerprints for *Klebsiella oxytoca* and *Klebsiella pneumoniae* species complexes (KoSC and KpSC, respectively) and co-occurring fecal bacterial strains from 10 preterm infants with NEC and 20 matched controls. Complementary approaches were used to identify cytotoxin-producing isolates of KoSC. *Klebsiella* species colonized most preterm infants, were more prevalent in NEC subjects versus controls, and replaced *Escherichia* in NEC subjects. Single KoSC or KpSC ASV fingerprinted strains dominated the gut microbiota, suggesting exclusionary *Klebsiella* competition for luminal resources. *Enterococcus faecalis* was co-dominant with KoSC but present infrequently with KpSC. Cytotoxin-producing KoSC members were identified in most NEC subjects and were less frequent in controls. Few *Klebsiella* strains were shared between subjects. We conclude that inter-species *Klebsiella* competition, within an environment of KoSC and *E. faecalis* cooperation, appears to be an important factor for the development of NEC. Preterm infants seem to acquire *Klebsiella* primarily through routes other than patient-to-patient transmission.

## Introduction

Necrotizing enterocolitis (NEC) is the most common and lethal gastrointestinal emergency of premature infants^1^. The premature intestine is uniquely hyper-responsive to pathogen-associated molecular patterns (PAMPS)^2^, which likely explains the destructive inflammatory response observed in this disorder. Along these lines, it is generally accepted that NEC pathogenesis involves activation of Toll-like receptor 4 (TLR4) signaling cascades by lipopolysaccharides (LPS) from the outer membranes of gut colonizing Gram-negative bacteria^3,4^. Whether colonization with particular Gram-negative bacteria is a feature of the dysbiotic microbial profiles that incite this runaway pro-inflammatory response remains an open question.

Gram-negative bacteria belonging to the genus *Klebsiella* have been linked to NEC in several studies, but methods of analysis often failed to discriminate *Klebsiella* species^5–7^. Most NEC microbiome studies have used 16S rRNA sequencing, which surveys complex microbial communities but typically lacks the depth to classify bacteria at the species or even genus levels. Members of the genus *Klebsiella* are highly diverse and include the *Klebsiella oxytoca* and *Klebsiella pneumoniae* species complexes (KoSC and KpSC, respectively) along with several more genetically distant species^8,9^. We previously employed a combination of 16S rRNA sequencing, selective culture systems, polymerase chain reaction and mass spectrometry to identify cytotoxin-producing *K. oxytoca* in the fecal microbiota of premature infants with NEC^10^. Toxigenic strains of KoSC produce the enterotoxins tilivalline and tilimycin and are the causative agent of antibiotic-associated hemorrhagic colitis (AAHC) in older children and adults^11–13^. Alternate approaches, such as genome-resolved metagenomics, have similarly identified *Klebsiella* as dominating the fecal microbiome of NEC subjects, while suggesting that strains of *K. pneumoniae* were more often associated with the disease^14^. Improved resolution of intestinal microbiota would help to clarify the potential role of different *Klebsiella* species and strains in the preterm gut.

Advances in sequencing platforms have greatly improved the accuracy of long-read, high-throughput approaches for surveying microbial communities^15^. We recently described a novel ~ 2500 base rRNA (StrainID) amplicon spanning the bacterial 16S and 23S rRNA genes that maps to a new, custom 16S–23S rRNA database to achieve species and strain level resolution of the gut microbiome in preterm infants^16^. The StrainID amplicon contains sufficient sequence variability such that a single *Klebsiella* strain can produce up to 8 different amplicon sequence variants (ASVs) which, when combined, create a unique fingerprint. Differentiation of closely related *Klebsiella*, *E. coli* and *Enterobacter* with this method enabled ASV fingerprinting to track specific strains in the hospital environment^16^. In the present study, we used this novel deep-sequencing approach to obtain evidence that colonization of the premature infant gut with either KpSC or KoSC strains appears to be a common precursor to the onset of NEC.

## Results

### Matched pairs used for analysis

During the study period, fecal samples from 10 preterm infants who developed NEC were available for analysis; samples from 20 matched controls were also analyzed. There were no significant differences in clinical characteristics between the two groups (Table 1). Adequate DNAs were extracted from 143 (44 NEC and 99 control) time points.

**Table 1:** Baseline characteristics of the subjects.

Characteristic	NEC (N = 10)	Control (N = 20)	*P* value
Birthweight (g)	817 (778–1125)	820 (720–1092)	0.681
Gestational age at birth (weeks)	25.6 (24.7–27.8)	25.6 (24.9–28.5)	0.940
Sex			0.333
Girls	1 (10)	5 (25)	
Boys	9 (90)	15 (75)	
Race			0.310
Black	3 (30)	8 (40)	
White	3 (30)	9 (45)	
Other	4 (40)	3 (15)	
Apgar scores			
1 min	5 (1.5–6.8)	3.5 (2.0–5.5)	0.701
5 min	7 (7.0–8.0)	8 (6.0–8.0)	0.813
Number of singleton births	10 (100)	18 (90)	N/A
Number of births by caesarean delivery	7 (70)	14 (70)	1.000
Age of necrotizing enterocolitis (days)	27 (19.3–34.0)	N/A	N/A
Percentage of days on human milk	70 (64.0–95.0)	91 (73.8–97.0)	0.737
Days of antibiotics prior to NEC or sample	4 (2.3–7.5)	5 (3.0–8.0)	0.558
Age first stool analyzed (days)	12.5 (8.3–15.5)	10 (7.0–14.3)	0.610
Number of stools analyzed	5 (3.0–5.8)	5 (3.0–6.0)	0.621

Data are median (interquartile range) or n (%); *NEC* necrotizing enterocolitis, *N/A* not applicable.

### *Klebsiella* species colonized most preterm infants, were more prevalent in NEC subjects versus controls, and replaced *Escherichia* in NEC subjects

**Figure 1:**
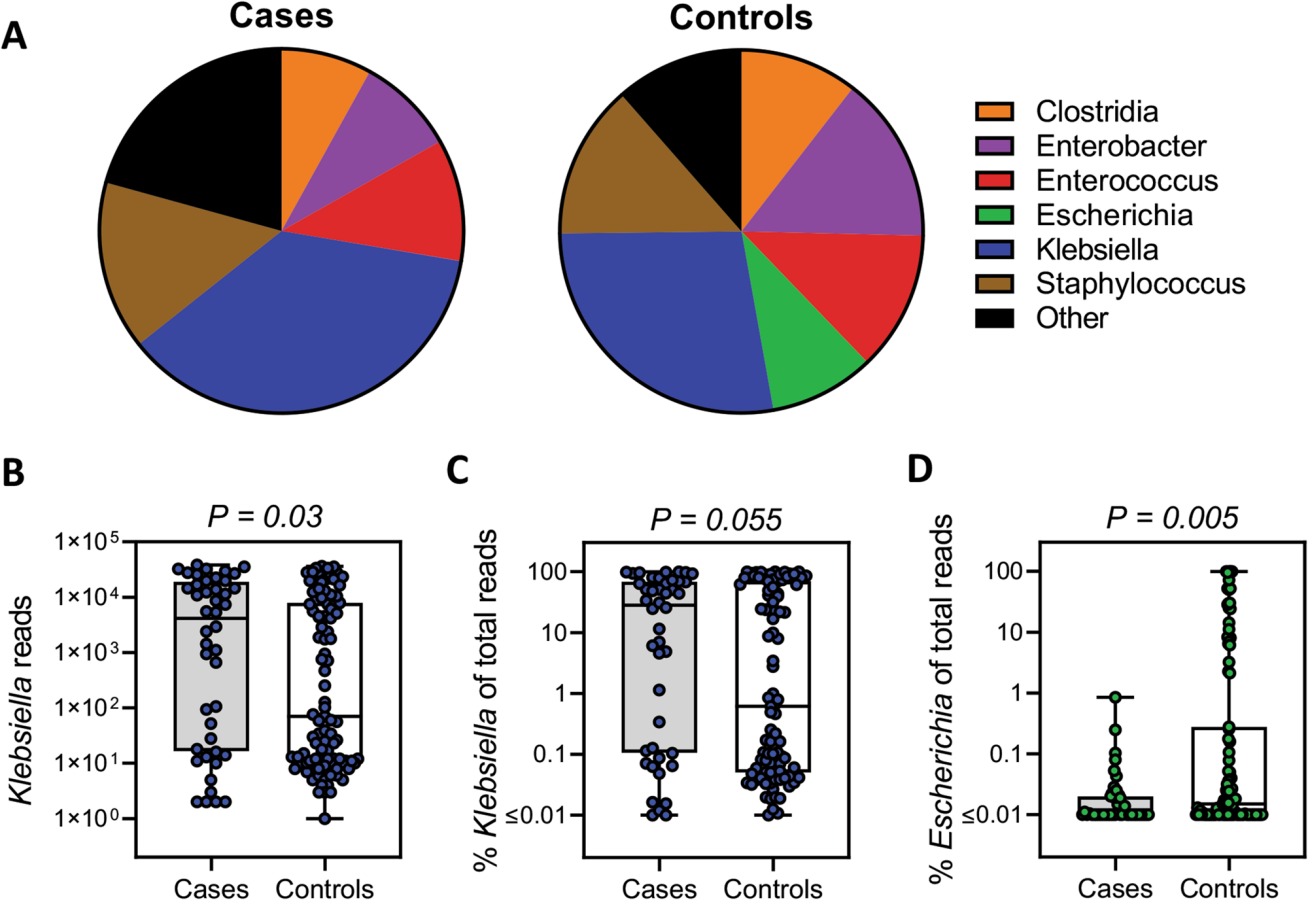
*Klebsiella* species dominated the gut microbiota of preterm infants and were more prevalent in NEC cases versus controls. (**A**) Distribution of the major genera in cases and controls. Total number of *Klebsiella* reads (**B**)*,* the percent *Klebsiella* of total reads (**C**), and the percent *Escherichia* of total reads (**D**), in cases and controls. The data are presented as mean values (**A**), or box plots of all points (**B**–**D**) with center lines representing medians, box limits indicating upper and lower quartiles, and whiskers spanning minimum to maximal values. Statistical analysis was by the Mann–Whitney U test.

StrainID amplicon sequencing returned a total of 3,462,451 reads with a mean of 24,213 ± 1443 reads per sample. *Klebsiella* was the predominant genus identified for both cases and controls, accounting for a combined 30.6% of total reads from the two groups (Fig. 1A). In individual samples, the mean number of *Klebsiella* reads was 10,181 ± 1777 and 6100 ± 1007, respectively, in the NEC and control groups (Fig. 1B; *P* = 0.03). When normalizing to total reads, *Klebsiella* accounted for 36.7 ± 5.6% of reads in the NEC group and 27.7 ± 3.7% of reads in the control group (Fig. 1C; *P* = 0.055). For individual subjects, *Klebsiella* was present at a relative abundance of > 2% in 9 of 10 NEC cases, and in 8 of them the relative abundance was > 7%. Alternatively, *Klebsiella* was present at a relative abundance of > 2% in 12 out of 20 controls, and in 10 of them the relative abundance was > 7% (*P* = 0.09 and *P* = 0.11 for comparisons at > 2% and > 7%, respectively). Additionally, the relative abundance of *Escherichia* was 9.5 ± 2.4% in the control group and 0.04 ± 0.02% in the NEC group (Fig. 1D; *P* = 0.005). There were no significant differences for other major taxa including *Enterococcus*, *Enterobacter*, *Clostridia*, *Veillonella*, *Staphylococcus* and *Streptococcus* between the groups (Fig. 1A).

### Differentiation of *Klebsiella* species revealed exclusionary competition between members of KoSC and KpSC

**Figure 2:**
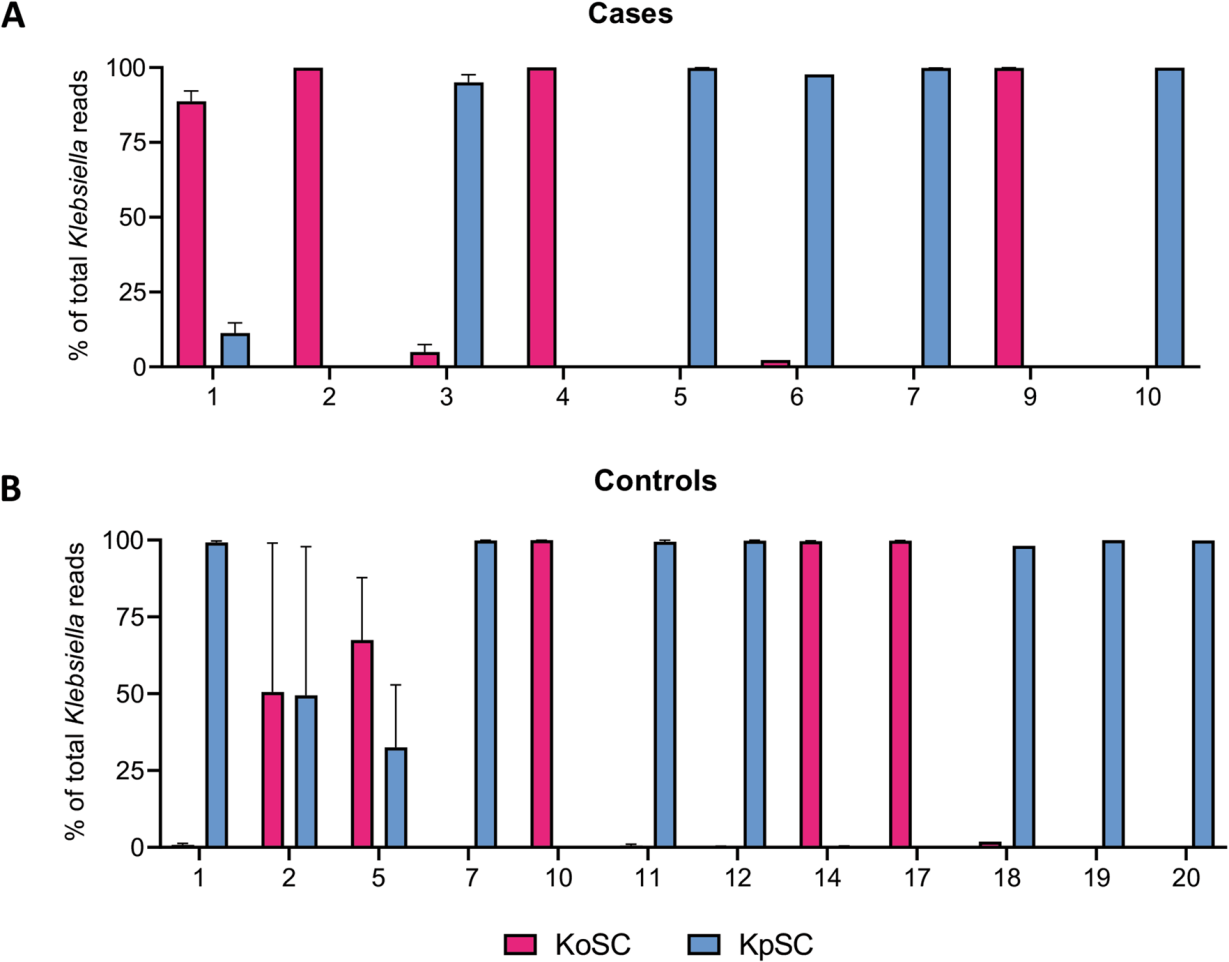
Exclusionary competition between KoSC and KpSC in the gut microbiota of preterm infants. The percent of *Klebsiella* reads corresponding to KoSC or KpSC in cases (**A**) and controls (**B**). The data shown are the composite profiles for all subjects harboring *Klebsiella* at > 2% relative abundance and are presented as mean values ± standard error of the mean. *KoSC* *Klebsiella oxytoca* species complex, *KpSC* *Klebsiella pneumoniae* species complex.

We next sought to use StrainID fingerprinting to differentiate *Klebsiella* species in our samples. All subjects that harbored *Klebsiella* at a relative abundance of > 2% (9 NEC and 12 controls) were used in the analysis. By plotting the composite relative abundance of reads assigned to *Klebsiella* species for each subject, we observed that 4 of 9 NEC cases were heavily dominated by KoSC, while the remaining five were heavily dominated by KpSC (Fig. 2A). A similar pattern of KoSC or KpSC dominance was found in the controls (Fig. 2B), and in subjects located at both NICU sites. KoSC and KpSC were present together in appreciable amounts in only 4 of 21 subjects (Cases 1 and 3, Controls 2 and 5). Among the other major genera identified, the species-level assignments for all subjects were as follows: *Staphylococcus* was 99% *S. epidermidis; Enterococcus* was 98% *E. faecalis*; *Clostridia* was 85% *C. perfringens* and 11% *C. difficile*; *Escherichia* was 61% *E. coli* and 39% *E. fergusonii*; and, *Enterobacter* was 27% *E. cloacae, 25% E. asburiae, 21% E. hormaechei,* along with several less prevalent species.

**Figure 3:**
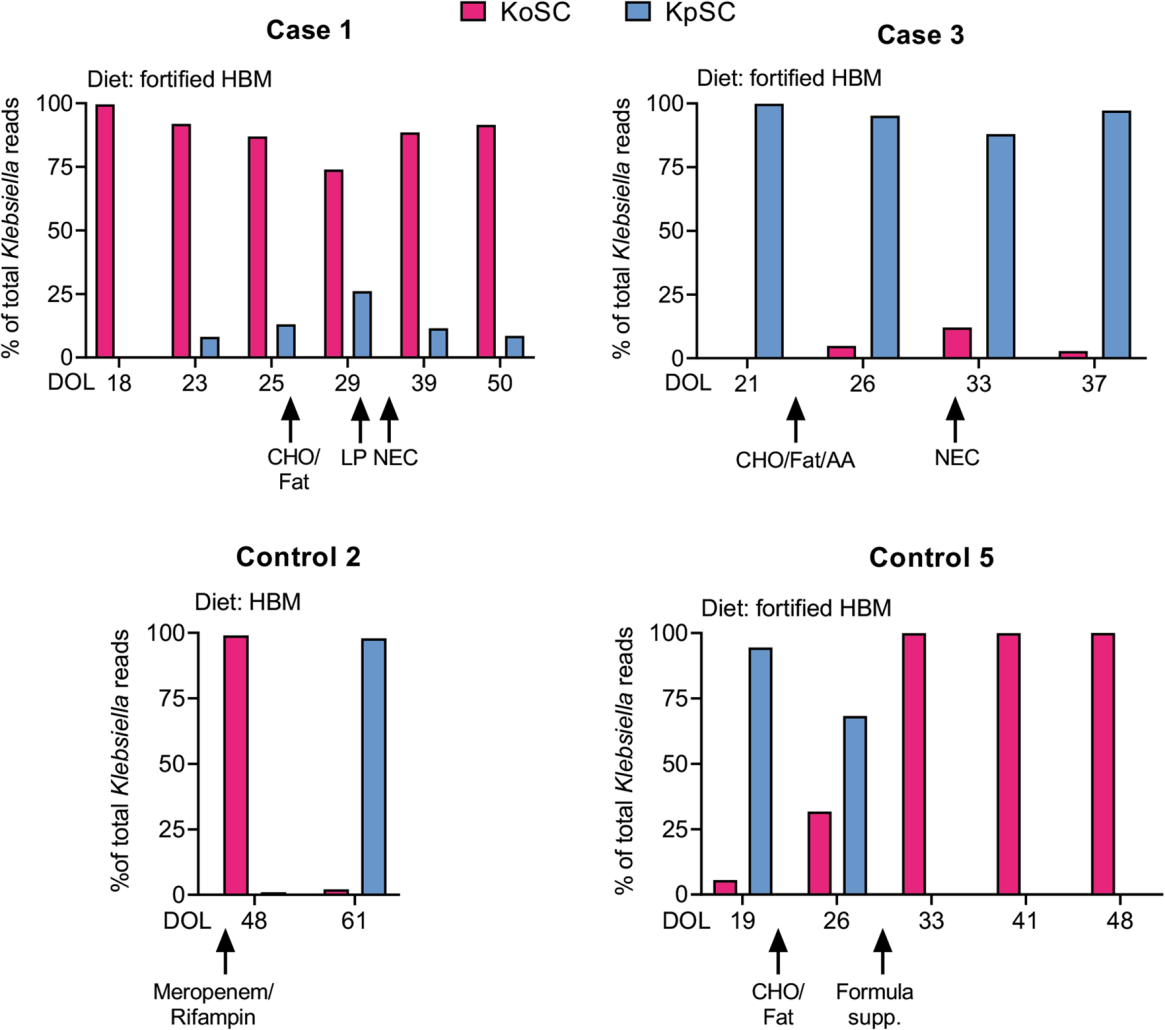
Fecal patterns of KoSC or KpSC dominance in relation to clinical factors. Shown are the percent of *Klebsiella* reads corresponding to KoSC or KpSC sequentially over time (Days of Life, DOL) for the 4 subjects harboring appreciable amounts of both species complex (Fig. 2). Arrows indicate the DOL for each occurrence. *KoSC* *Klebsiella oxytoca* species complex, *KpSC* *Klebsiella pneumoniae* species complex, *HBM* human breast milk, *CHO/Fat* carbohydrate/fat powder, *LP* liquid protein, *AA* amino acid powder, *NEC* necrotizing enterocolitis.

To further clarify the relationship between KoSC and KpSC in the 4 subjects with appreciable amounts of both, we determined their relative abundances in successive fecal samples over time and in relation to clinical parameters (Fig. 3). Case 1 demonstrated a pattern of KoSC dominance throughout successive weeks despite detection of low levels of KpSC at later time points; no antibiotics were administered during the time points prior to the development of NEC. Enteral feeds were fortified human milk and the diet was supplemented with carbohydrate/fat powder and liquid protein 8 and 4 days, respectively, before the development of NEC. Case 3 displayed the reverse pattern, with KpSC dominance despite detection of low levels of KoSC in later weeks; no antibiotics were administered during the time points prior to the development of NEC. Enteral feeds were fortified human milk and the diet was supplemented with carbohydrate/fat plus amino acid powder 8 days before the development of NEC. Controls 2 and 5, on the other hand, demonstrated dramatic shifts between KoSC and KpSC over successive weeks. Control 2 received treatment with Meropenem and Rifampin for a non-intestinal infection during the interval period but no dietary changes. Control 5 was receiving feeds of fortified human milk and the diet was supplemented with carbohydrate/fat powder 5 days before an increase in KoSC, and with formula 4 days before the disappearance in KpSC; no antibiotics were provided during the time points.

### StrainID amplicon sequencing demonstrated that very few *Klebsiella* strains were shared between subjects

**Figure 4:**
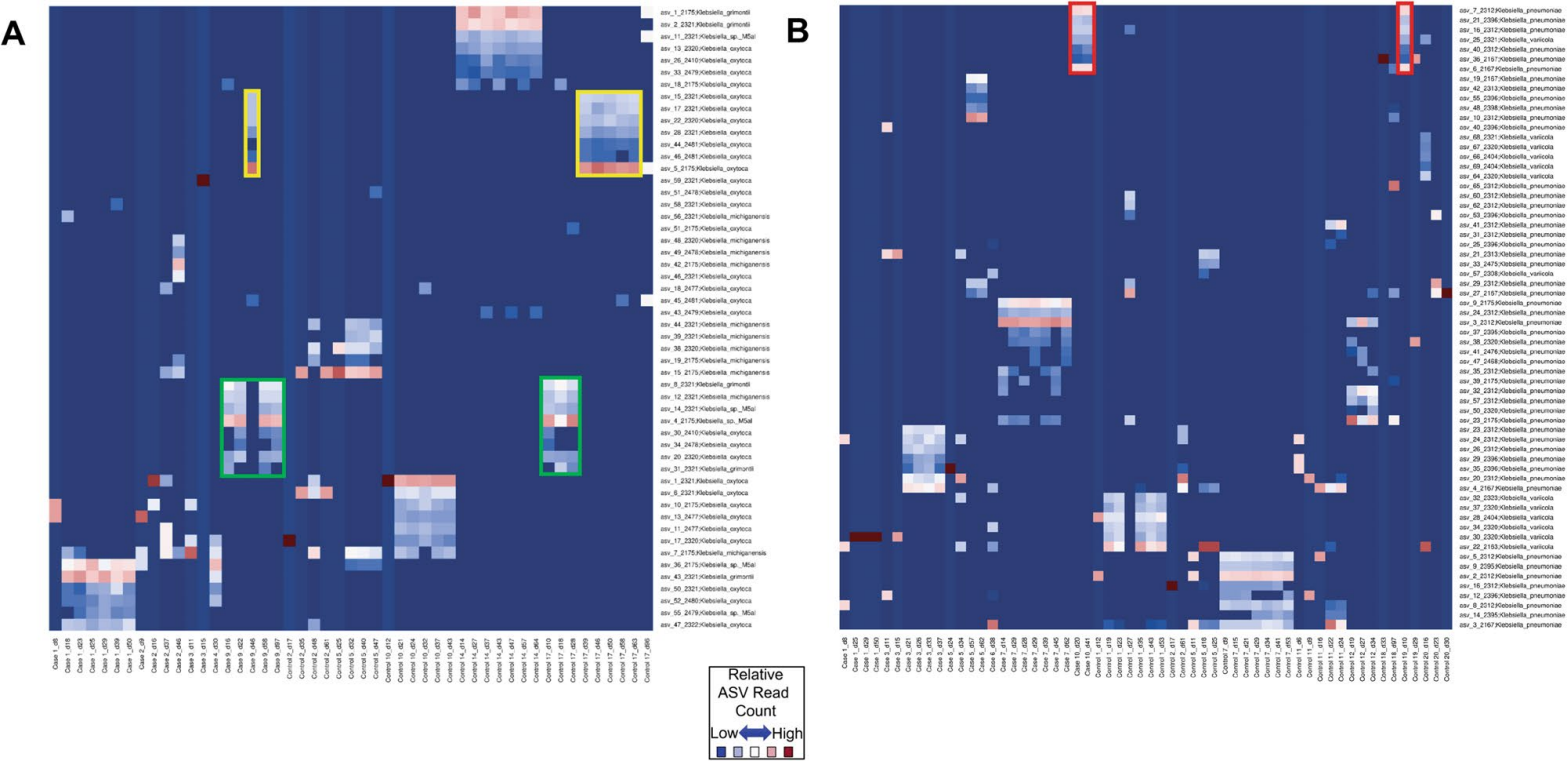
Amplicon sequence variant (ASV) fingerprints demonstrated that very few KoSC and KpSC strains were shared between subjects. Relative-abundance heat maps of StrainID ASVs classified as either KoSC (**A**) or KpSC (**B**); samples sharing strains have similar ASV fingerprint patterns^16^. On the X axes, samples from each subject are denoted with (d) corresponding to the day-of-life of sample collection. Species-level taxonomic assignments for the ASVs are indicated on the Y axes. Each ASV in a sample was colored according to the relative number of reads. (**A**) ASV fingerprint patterns outlined in yellow and green indicate the subjects were likely colonized with the same two KoSC strains. (**B**) ASV fingerprint patterns outlined in red indicate subjects likely colonized with the same KpSC strain.

To differentiate the *Klebsiella* strains involved, we analyzed the StrainID amplicons using DADA2 to infer distinct ASV groups that correspond to individual strains^16^. This approach takes advantage of the fact that each individual *Klebsiella* genome contains eight copies of a 16S-ITS-23S rRNA operon that can vary in sequence and length. The eight copies produce eight distinct amplicons for a single genome, which vary in length and sequence. Generating multiple amplicons per genome presents the opportunity to use the combination as a ‘fingerprint’ profile to identify a given strain, even if closely related strains share one or more 16S–23S variants. ASV fingerprint patterns representing distinct KoSC or KpSC strains were present in all 9 NEC cases and 12 controls (Fig. 4A,B). The finding that some ASV groupings contained less than eight ASVs indicates that some strains may harbor duplicate copies^16^; alternatively, ASVs located distant from the origin of replication or that contain a longer ITS region (e.g., more tRNA genes) might drop off before others. Nevertheless, the ASV fingerprinting patterns were distinct and most strains among the NEC cases and controls were unique, indicating that transmission of dominant strains within the subject populations was not occurring. Case 9 and Control 17 shared two distinct patterns, indicating they were likely colonized with the same KoSC strains at different time points (Fig. 4A), while case 10 and Control 19 shared patterns indicating they were likely colonized with the same KpSC strain (Fig. 4B).

### Cytotoxin-producing KoSC members were more prevalent in NEC subjects versus controls

Members of KoSC can harbor a biosynthetic gene cluster responsible for generating the enterotoxins tilimycin and tilivalline^12^, which may facilitate mucosal damage in NEC^10^. To determine if cytotoxin-producing KoSC was more prevalent in NEC cases versus controls, and in KoSC- versus KpSC-dominated microbiomes, we analyzed stool samples using PCR and a selective culture system as described in “Methods”. Toxin-positive KoSC members were identified and cultured from 7 of 10 NEC cases and 4 of 20 controls (P = 0.007) (Table 2; Supplementary Tables S1, S2; Figure S1). Phylogroup assignment of the KoSC isolates demonstrated *K. michiganesis* and *K. grimontii* to be dispersed across both NICU sites; whereas, *K. oxytoca* was localized to one NICU (Table 2). In one control subject (Control 2), KoSC was not detected by PCR or culture, despite KoSC reads being present in the StrainID analysis; this subject received broad-spectrum antibiotics during specimen collection. In all remaining subjects, KoSC isolates were recovered when StrainID identified appreciable KoSC reads. Toxin-positive KoSC strains were detected in several KpSC-dominated microbiomes of NEC subjects (Cases 3, 5 and 10) and in one NEC subject (Case 8) with a low (< 2%) overall abundance of *Klebsiella*. Toxin-positive KoSC was identified in one control (Control 20) with a KpSC-dominated microbiome; this infant was treated for suspected (Bell’s stage 1), but not confirmed, NEC. No KoSC strains were identified in controls with a low (< 2%) overall abundance of *Klebsiella*. Collectively, these data demonstrate the greater prevalence of cytotoxin-producing KoSC in NEC cases compared to controls; KoSC was either the dominant *Klebsiella* species or secondary to KpSC in the majority of NEC cases*.*

**Table 2:** KoSC isolates recovered from cases and controls.

Subject	Taxonomic assignment	Toxin +/−	NICU site
Case 1	*K. grimontii*^*a,b*^	+	1
Case 2	*K. michiganesis*^*a*^	−	1
Case 3	*K. michiganesis*^*a*^	+	2
Case 4	*K. grimontii*^*a*^ *K. michiganesis*^*a*^	+ −	2 2
Case 5	*K. grimontii*^*a*^ *K. michiganesis*^*a*^	+ -	1,2^c^ 1,2^c^
Case 8	*K. oxytoca*^*b*^	+	1
Case 9	*Unclassified KoSC*^b^*	+	2
Case 10	*K. grimontii*^*b*^	+	1,2^c^
Control 5	*K. michiganesis*^*a*^	−	1
Control 10	*K. michiganesis*^*a*^	+	1
Control 14	*K. grimontii*^*b*^	+	2
Control 17	*K. oxytoca*^*b*^	+	1
Control 20	*Unclassified KoSC*^b^*	+	1

Toxin +/− denotes the presence or absence of *npsA/B*. ^a^Taxonomic assignment by average nucleotide identity (ANI)/OrthoANI^47^. ^b^Taxonomic assignment by Cosic et al. PCR scheme^22^. *Maps closest to *K. grimontii.* *KoSC* *Klebsiella oxytoca* species complex, *K Klebsiella.* NICU site indicates the caring facility. ^c^Received care at both facilities.

### *Enterococcus faecalis* was co-dominant with KoSC but present infrequently with KpSC

**Figure 5:**
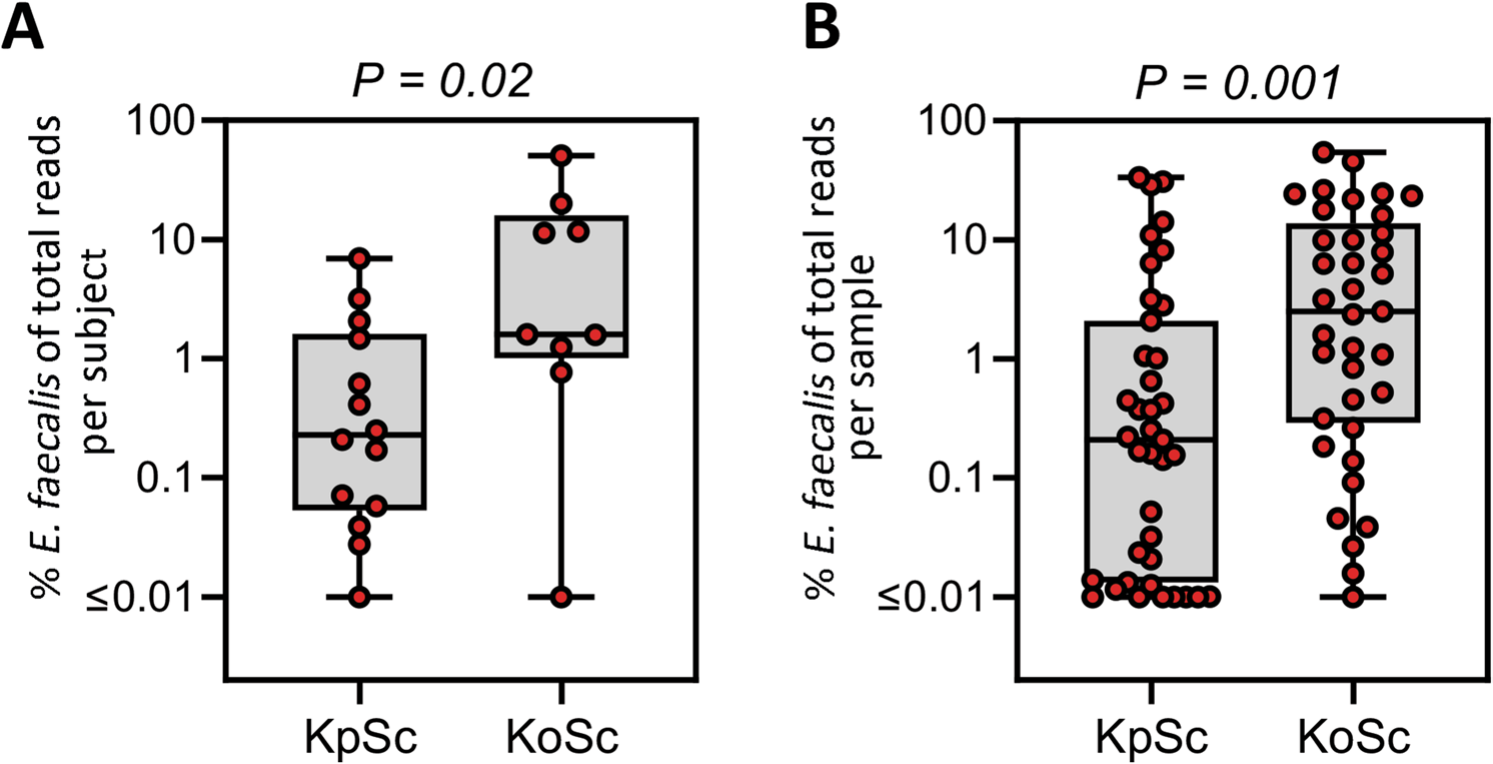
*Enterococcus faecalis* was frequently co-dominant with KoSC but not with KpSC. The relative abundance of *E. faecalis* was determined in fecal samples dominated by KoSC or KpSC. Shown are the differential abundances per subject (**A**) and per sample (**B**). The data are presented as box plots of all points with center lines representing medians, box limits indicating upper and lower quartiles, and whiskers spanning minimum to maximal values. Statistical analysis was by the Mann–Whitney U test. *KoSC* *Klebsiella oxytoca* species complex, *KpSC* *Klebsiella pneumoniae* species complex.

To ascertain if other bacteria were associated with KoSC or KpSC, the prevalence of other microbes was determined by StrainID. The analysis revealed *E. faecalis* to be co-dominant with KoSC, but not with KpSC. On a per subject basis, *E. faecalis* accounted for 11% and 1% of the reads, respectively, in KoSC- and KpSC-dominated microbiomes (P = 0.02, Fig. 5A). On a per sample basis, the co-dominant pattern of *E. faecalis* and KoSC vs. KpSC was more pronounced (P = 0.001, Fig. 5B). No significant associations were found between KoSC or KpSC and the abundance of *Clostridia*, *Staphylococcus*, *Veillonella*, *Enterobacter* or *Escherichia *spp*.*

## Discussion

A substantial body of evidence points to LPS-induced TLR4 signaling cascades as causal to the profound intestinal inflammatory response observed in infants with NEC^3^. While gut dysbiosis with Gram-negative bacteria is believed to be a key antecedent event^4,17^, there is uncertainty as to whether gut colonization with particular bacterial species predisposes to the development of NEC. In this regard, it is unlikely that all colonizing Gram-negative bacteria share the same pathogenic potential to incite intestinal damage^18^. Members of the genus *Klebsiella* have been implicated in previous studies^5–7^; however, specific species identification is generally not possible using phenotypic characteristics or short segments of the 16S rRNA gene (e.g., V4)^9^. Here, we utilized a novel long-read amplicon spanning the 16S and 23S rRNA genes to determine the relative abundance of KoSC and KpSC across NICU subjects at risk for developing NEC. We found that *Klebsiella* was ubiquitous, colonizing the majority of preterm infants; consistent with previous reports^5–7,10,14^, *Klebsiella* was more prevalent in NEC subjects compared to non-NEC controls. We further reveal a pattern of colonization in which the gut microbiota was heavily dominated by either KoSC or KpSC, suggesting competition between the two for luminal resources. The data provide new insights into the microbial community structure in the preterm gut and how members of the genus *Klebsiella* may contribute to this catastrophic outcome of prematurity.

*Klebsiella* spp. are opportunistic pathogens that are frequently isolated from hospital environments and are a leading cause of nosocomial infections^8,9^. The healthy human microbiome also serves as a potential reservoir for infection; gut colonization with *Klebsiella* spp. is a well-established precursor for extraintestinal infection in adults^19,20^. While *Klebsiella* spread among adults is often multi-strain^19,20^, single strain outbreaks also occur^21^. Our analysis of *Klebsiella* ASV fingerprint patterns identified specific strains in the fecal microbiota of preterm infants and demonstrated that very few KoSC or KpSC strains were shared between subjects. Partial overlap of ASVs between closely related strains and even phylogroups can occur^16^ which reflects taxonomic similarities within the 16S–23S region for each species complex. The ASV fingerprinting enables tracking of individual strains, but whole genome sequencing or PCR for genes outside of the 16S–23S region are necessary to further classify KoSC and KpSC members^22,23^. Despite partial overlap of some ASVs, the fingerprint patterns were distinct and recurring for those harboring the same KoSC and KpSC strain. The absence of dominant strains argues against there being a common environmental reservoir (e.g., sink, bedding). We did not sample parents in our cohort, and, given the broad distribution of strains, it is possible that parental transmission or alternate reservoirs were involved.

Production of the cytotoxins tilimycin and tilivalline by KoSC can cause AAHC in older children and adults^11–13^ and has been linked to NEC in premature infants^10^. Our analysis detected cytotoxin-producing KoSC in the majority of NEC subjects. Surprisingly, several toxin-producing isolates were recovered from NEC subjects whose gut microbiomes were dominated by KpSC*.* The pattern of either KoSC or KpSC dominance suggests exclusionary *Klebsiella* species competition, and the context in which competitive stress could increase the expression of virulence factors^24^ warrants further investigation. It is possible that intestinal injury represents collateral damage as the result of competitive mechanisms unleashed during *Klebsiella* inter-species warfare. In the preterm gut, these processes may be further exacerbated by activation of TLR-4 signaling pathways in response to *Klebsiella* LPS^2,3,25,26^. Thus, the culmination in the development of NEC could involve competitive microbial communities interacting with bacterial signaling receptors (e.g., TLR4) on the premature intestine.

Members of KoSC utilize a broader array of sugars than KpSC which enables them to outcompete KpSC in murine models of gut colonization^27^, while providing the fermentative energy to support cytotoxin production^28^. In Case 3 and Control 5, an increase in KoSC abundance occurred following an increase in dietary carbohydrates. The development of NEC also occurred in Cases 1 and 3 after an increase in dietary carbohydrates. Preterm infants are at risk for carbohydrate malabsorption because they have under-developed intestinal brush border enzymes^29^. The availability of undigested carbohydrates may serve as the impetus for KoSC and KpSC to engage each other in conflict over valuable resources.

Provision of broad-spectrum antibiotics enhances colonization with KpSC^30^. Along these lines, the dramatic shift in KoSC to KpSC observed in Control 2 corresponded with antibiotic treatment for a non-intestinal infection. Many members of KpSC also exploit the type VI secretion system (T6SS) which can facilitate intra- and inter-species killing depending on environmental cues^31^. Deciphering the contextual factors in the gut in association with diversity of genes regulating sugar utilization, cytotoxin production, anti-microbial resistance and T6SS would assist in establishing predictive patterns of KoSC versus KpSC dominance.

*Escherichia* was found to be significantly higher in the non-NEC controls, which could indicate that members of this taxa filled a niche that was occupied by *Klebsiella* in the cases. Enhanced sugar utilization by members of KoSC is known to facilitate colonization resistance against *E. coli*^32^; thus, an alternate explanation is that *Escherichia* was replaced by *Klebsiella*. Others have observed an increase in *E. coli* prior to the onset of NEC^33^, and that specific strains of *E. coli* are associated with more severe disease compared to *Klebsiella*-associated NEC^34^. Notably, Ward and colleagues found that *E. coli* and *Klebsiella* were the two most abundant species in a cohort of preterm infant with NEC; however, the two were infrequently co-habitants^34^. The outcome of competitive interactions among and between *E. coli* and *Klebsiella* are likely dependent on the genetic characteristics of individual strains. At the phylum level, microbial dysbiosis preceding NEC is frequently characterized by an increase in *Proteobacteria* and decrease in *Firmicutes*^35^. Nevertheless, some reports have demonstrated that early colonization with *Firmicutes*, such as *Staphylococcus* and *Enterococcus,* enhance the risk of developing NEC at later time points^17,36^. We did not observe any associations between *Firmicutes* and the development of NEC. The abundance of *Bifidobacterium* spp. also was very low among the subjects, which is consistent with reports of these communities being uncommon in very and extremely premature infants^37^.

Several reports have linked colonization of *Enterococcus* with KpSC^38,39^. In the current study, we found that *E. faecalis* was predominantly associated with KoSC, rather than KpSC. Recently, a broad scale genomic analysis differentiated KoSC from KpSC by the presence of genes involved in a type II system to secrete pullulanase, a debranching enzyme that breaks down complex sugars^40^. *Enterococci* do not express glycosidases that degrade mucosal polysaccharides, and their carbohydrate utilization is limited to less complex sugars^41^. Therefore, it is possible that *E. faecalis* co-colonizes with KoSC via cooperative carbohydrate metabolism^42^, whereby *E. faecalis* takes advantage of pullulanase converting complex polysaccharides into small fermentable sugars. Cytotoxin production by KoSC also may be contributory as tilimycin was recently reported to enhance *Enterococcus* growth and restrict *E. coli* colonization^43^.

In summary, StrainID amplicon sequencing and ASV fingerprinting provides improved resolution to differentiate KoSC and KpSC in fecal samples and suggests that the two species are in direct competition in the preterm gut. Untangling the contextual factors, genetic diversity of specific strains and competitive mechanisms that can result in intestinal injury are important to understand how these microbes contribute to this devastating disease of prematurity.

## Methods

### Study population

Subjects were cared for at two affiliated NICUs in Hartford and Farmington, CT. Infants with a gestational age of less than 32 weeks were enrolled from March 2017 to October 2019; those with known congenital malformations of the intestine or not expected to survive beyond the first week were excluded. Study subjects underwent routine care and informed written consent was obtained from a parent on behalf of their infant. Cases were infants whose clinical courses and radiographic findings were consistent with Bell’s stage 2 or 3 NEC^44^. Two control subjects were matched to each NEC case. The study was approved by the Institutional Review Board of Connecticut Children’s Medical Center and all research was performed in accordance with relevant guidelines/regulations.

### Collection of samples and clinical data

Fecal samples were collected on an approximate weekly basis using sterile disposable spatulas during diaper changes, placed into sterile containers, and immediately frozen at − 80 °C until processing. Samples collected prior to and up to 2–3 weeks after the diagnosis of NEC were included in the analysis. Samples from control infants were time-matched by the closest chronological age corresponding to case samples. Clinical data were obtained from enrolled infants including demographics, gestational age, mode of delivery, day of life (DOL) of NEC, DOL of sample acquisition, exposure to antibiotics, and diet.

### StrainID amplicon sequencing

Fecal samples were processed and analyzed as previously described^16^. Briefly, fecal DNA was purified, PCR amplified, and pooled for sequencing using the Complete StrainID Kit (StrainID set A [barcodes 1 to 96]; Intus Biosciences, Farmington, CT) according to the manufacturer’s instructions. Amplicon libraries were created using the SMRTbell express template prep kit 2.0 (catalog number 100-938-900; PacBio). The library was sequenced on a Sequel IIe system (Pacific Biosciences) at the University of Delaware, Delaware Biotechnology Institute Sequencing and Genotyping Center, Newark, DE. The selected reads from each sample were primer trimmed and filtered to reads within the length range of 1900–3000 bp. The trimmed and filtered reads were analyzed manually via a histogram to identify peaks of read lengths that are likely to represent unique amplicons. The corresponding read length ranges (i.e., the 2400- to 2405-bp range from each sample) were passed to DADA2^45^ and pooled for ASV inference^16^. A sequence table of ASV abundance per sample was produced as part of the DADA2 output, and a heat map was generated in R using the sequence table. SBanalyzer 2.4 (Intus Biosciences) was used to map ccs reads to the Athena database and assign taxonomic identification^16^.

### Identification of cytotoxin-producing KoSC in fecal samples

DNA from fecal samples and clinical isolates was extracted using the DNeasy Power Soil Kit and DNeasy Blood and Tissue Kit (Qiagen, Germantown, MD), respectively. Fecal samples were screened by PCR for the KoSC-specific gene *pehX* and genes encoding enzymes in the cytotoxin biosynthetic pathway (*npsA* and *npsB*)^46^. For culturing KoSC, a loopful of fecal material was inoculated into 5 ml of Luria–Bertani (Lennox) broth (LB) and grown for 24 h with shaking at 225 rpm at 37 °C. Serial dilutions of fecal cultures were plated on hydroxybenzoic agar as previously described^10^. Individual KoSC colonies were confirmed as toxin-positive or -negative by PCR (*pehX*, *npsA/B*)^10^. For taxonomic assignments of the isolates, libraries were made using NexteraXT and sequenced on MiSeq v2 500 cycle (Illumina Inc., San Diego, CA) at UConn Microbial Analysis, Resources, and Services, Storrs, CT. The sequenced genomes were compared across various KoSC phylogroups and an average nucleotide identity (ANI)/OrthoANI value of 98% or greater indicated a matching phylogroup^28,47^. Isolates that were not analyzed by (ANI)/OrthoANI were classified using a PCR-typing strategy to detect the following genes: *npsA*, *bla*_OXY-1_, *bla*_OXY-2_, *bla*_OXY-4/6_, *orf*ABC, *leup*AB, and *orf*A’^22^. Isolates were further characterized by their ability to metabolize d-melezitose^48^. The primer sequences used were as follows: *npsA* 5′-GCGCTGTTATGGTTCCCGT-3′ and 5′-CCGGGCACGCTTGTTACATC-3′; *npsB* 5′-TGCAGGGTACGCTAAATATTTTAGCT-3′ and 5′-ACCCACTTACTTTGCGTATAACCAAT-3′; *pehX* 5′-GATACGGAGTATGCCTTTACGGTG-3′ and 5′-TAGCCTTTATCAAGCGGATACTGG-3′; all other primer sequences for the PCR-typing strategy are as published^22^.

### Statistics

The analyses were carried out using GraphPad Prism version 9.3.0 (GraphPad Software, San Diego, CA). Mann–Whitney U test was used for quantitative data and Chi-square test for categorical data. A minimum threshold of 400 reads per sample was used to reduce outliers in the microbiome analysis.

## Supplementary Information

Supplementary Information.

## Data Availability

StrainID amplicon data and the whole-genome assemblies for KoSC isolates are deposited under Bioproject accession number PRJNA908822.
