# Supplementary material for: High-resolution microbiome analysis reveals exclusionary Klebsiella species competition in preterm infants at risk for necrotizing enterocolitis
Source: Sci Rep. 2023 May 16;13:7893. doi: 10.1038/s41598-023-34735-2 (PMC10188488; doi:10.1038/s41598-023-34735-2)
Supplement: Supplementary file 1 — Supplementary Information. [file 41598_2023_34735_MOESM1_ESM.pdf]

Table S1: Taxonomic assignment of KoSC isolates by average nucleotide identity/OrthoANI.

	OrthoANI value [%]			
	K. oxytoca	*K. michiganensis*	*K. grimontii*	*K. pasteurii*
Case 1	91.34	93.61	**99.25**	96.13
Case 2	92.21	**98.74**	93.69	93.84
Case 3	92.29	**99.10**	93.63	93.83
Case 4_1	91.35	93.68	**99.25**	96.17
Case 4_2	92.30	**99.02**	93.68	93.83
Case 5_1	91.42	93.68	**99.30**	96.19
Case 5_2	92.39	**99.03**	93.75	93.86
Control 5	92.34	**98.95**	93.77	93.95
Control 10	92.30	**99.12**	93.69	93.76

Reference strains: *K. oxytoca* NCTC13727 (GCA_900636985.1), *K. michiganensis* CAV1374 (GCA_001022195.1), *K. grimontii* JKO3 (GCA_001548355.1) and *K. pasteurii* isolate SB6412 (CABGHC000000000.1).

Table S2: Taxonomic assignment of KoSC isolates by PCR and melezitose fermentation.

	npsA	bla_{OXY-1}	bla_{OXY-2}	$bla_{OXY-4/6}$	orf_{ABC}	*leupAB*	*orfA'*	melezitose	Strain
Case 1	+	+	-	+	+	-	-	-	*K. grimontii*
Case 8	+	-	+	+	-	-	-	+	*K. oxytoca*
Case 9	+	+	-	+	-	-	-	-	unclassified
Case 10	+	+	-	+	+	-	-	-	*K. grimontii*
Control 14	+	+	-	+	+	-	-	-	*K. grimontii*
Control 17	+	-	+	+	-	-	-	~	*K. oxytoca*
Control 20	+	+	-	+	-	-	-	-	unclassified
Pos. Control	+	+	-	+	+	-	-	-	*K. grimontii*

+ = positive, - =negative, ~ = weak.

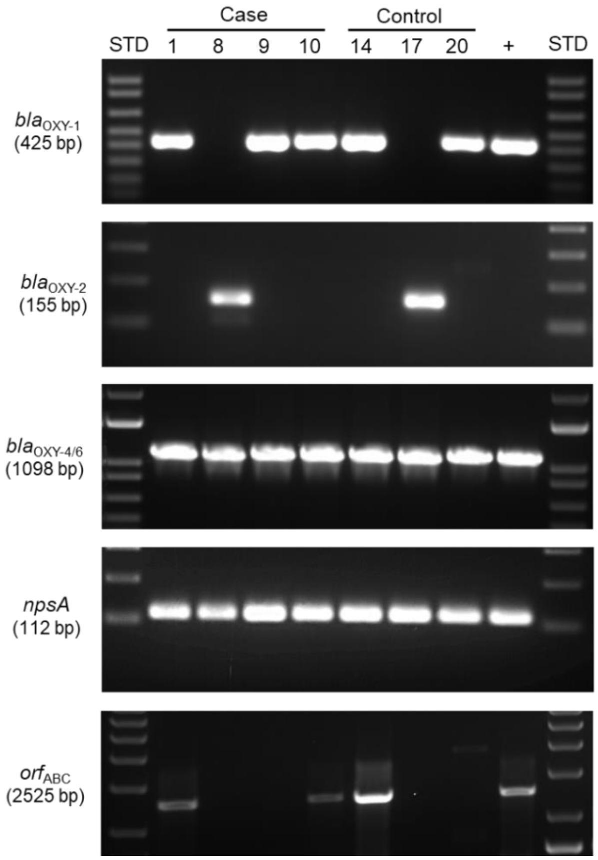

ST: 1 kb plus DNA ladder Invitrogen
Case 1: *K. grimontii*
Case 8: *K. oxytoca*
Case 9: unclassified
Case 10: *K. grimontii*
Control 14: *K. grimontii*
Control 17: *K. oxytoca*
Control 20: unclassified
+: pos. control, *K. grimontii* UCH-1

Figure S1. Genomic DNA from the fecal isolates was used as a template to perform PCR for the presence of bla_{OXY-1}, bla_{OXY-2}, $bla_{OXY-4/6}$, *npsA* and *orf*ABC. Phylogroup assignments were as described by Cosic et al. Cropped images are shown and represent the data presented in Table S2; original gels are presented in Figure S2.

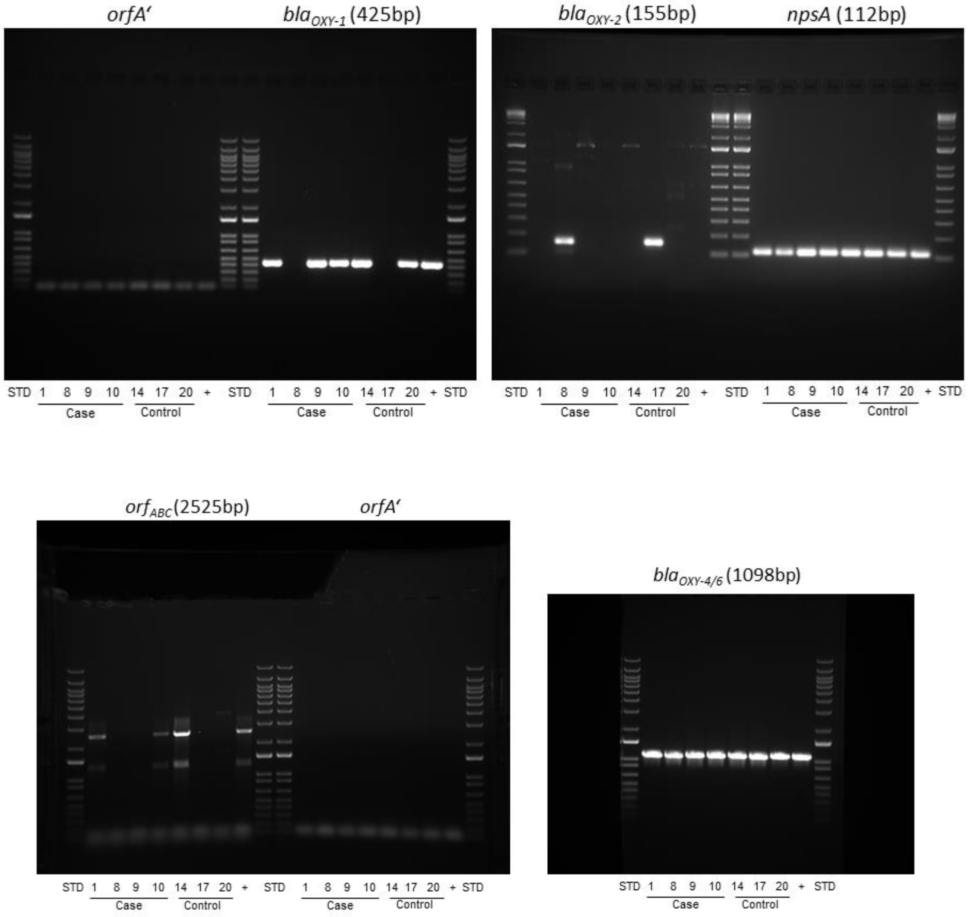

Figure S2. Uncropped gel images reflecting the data presented in Table S2 and Figure S1.
